# Electro-Blown Spun Ultra-High Molecular Weight Poly(L-Lactic Acid) Fibrous Membranes for High-Efficiency Air Filtration in Multiple Environments

**DOI:** 10.3390/nano16100604

**Published:** 2026-05-14

**Authors:** Hang Wang, Zhaoqing Wang, Yun-Ze Long, Wenpeng Han

**Affiliations:** 1 Shandong Key Laboratory of Medical and Health Textile Materials, College of Physics, Qingdao University, Qingdao 266071, China; wanghang2@qdu.edu.cn (H.W.); wangzhaoqing@qdu.edu.cn (Z.W.); 2 Innovation Institute for Advanced Nanofibers, Collaborative Innovation Center for Nanomaterials & Devices, Qingdao University, Qingdao 266071, China

**Keywords:** poly(L-lactic acid) fibrous membrane, ultra-high molecular weight, multiple environmental air filtration, sustainability, chemical stability

## Abstract

The bio-based biodegradable material poly(L-lactic acid) (PLLA) has received extensive attention due to its inherent sustainability. Ultra-high molecular weight (UHMW) PLLA possesses superior physical, chemical, and mechanical properties, but its difficulty in processing often restricts its further application. In this work, efficient preparation of UHMW PLLA fiber membranes using electro-blown spinning technology was reported for the first time. Thanks to the excellent electrostatic adsorption capacity brought by the piezoelectric properties of the prepared fiber membrane and its fluffy multi-scale structure, it demonstrates outstanding air filtration performance. The contradiction between filtration efficiency (>99.8% for PM_0.3_) and pressure drop (~16 Pa) has been successfully balanced. It also demonstrated excellent moisture resistance, long-term stability, and dust-holding capacity. Especially compared with low molecular weight PLLA fiber membranes, the air filtration performance of UHMW PLLA fiber membranes have demonstrated excellent chemical stability. Meanwhile, its temperature stability can also meet the needs of most scenarios in life. This ensures the feasibility of practical application of the sustainable material PLLA in the field of air filtration. And due to its excellent filtration performance, it reduces energy consumption during use, thereby achieving sustainable development throughout the material’s entire life cycle.

## 1. Introduction

Atmospheric particulate matters (PMs) not only can cause severe weather conditions such as smog and acid rain, but also can directly enter the body through the respiratory tract, triggering or aggravating diseases and affecting physical health [1,2,3]. Particularly, it can carry viruses and spread them, leading to large-scale outbreaks of infectious diseases and even affecting the world, such as the recent global outbreak of the novel coronavirus [4]. Therefore, the ways to reduce PM emissions, limit their spread range, and take good personal protective measures have all received widespread attention. To address these issues, especially the latter two, it becomes particularly important to prepare filter membranes with excellent filtration performance. During the filtration process, high filtration efficiency indicates extremely strong interception capacity for PMs, while a low pressure drop means low energy consumption during use and comfortable wearing for personal protection. Therefore, while improving filtration efficiency of a filter membrane, controlling the increase in pressure drop as much as possible has become the primary issue that relevant researchers need to solve. In addition, in practical applications, not only do industrial filtration scenarios often involve high temperatures, high humidity, and acidic or alkaline atmospheres, but the conditions in daily life are also constantly changing, and can even be harsh. For instance, with change in latitude and seasons, there are significant variations in temperature, and changes in air humidity are even more common. Spoilage and deterioration of a large amount of fruits and meats in large markets or municipal refuse disposal stations can also cause changes in acidity and alkalinity. Therefore, development and preparation of air filtration membranes with multiple environmental stabilizations is also a key focus of current scientific researchers.

Electrospun polymer material nanofiber membranes, due to their advantages, such as large specific surface area, high porosity, and adjustable structure, have broad application prospects in the field of air filtration [5,6]. Recently, they have received extensive research and made significant progress. Researchers successfully prepared a series of fiber membranes with special structures and fiber morphologies, such as spider web structures [7,8,9], multi-scale structures [10,11], as well as ribbon-like [12,13], beaded [14,15], and porous fibers [16,17], by adjusting the composition of the spinning solution, spinning parameters, and conditions. The contradiction between high filtration efficiency of the filter membrane and low pressure drop has been successfully reconciled. Meanwhile, some polymer materials themselves possess excellent high-temperature resistance, chemical stability, and other outstanding properties. Based on this, a series of electrospun air filtration membranes with excellent filtration performance capable of being used in harsh environments have been successfully prepared [18,19,20,21]. These research advancements have all demonstrated application potential of electrospun nanofiber membranes in the field of air filtration. Nevertheless, the existing nanofiber membranes still have many deficiencies: it is generally difficult to achieve long-term stable coexistence of efficient filtration and low pressure drop, structural stability and service durability under harsh environments are insufficient, and the relationship between the filtration mechanism, the structure, and performance has not been systematically revealed, making it difficult to meet the actual needs of multi-scenario adaptation and long-term service [22,23]. Furthermore, raw materials used in the above-mentioned research fields are mostly non-biodegradable materials from fossil sources. Their large-scale use will undoubtedly cause environmental problems and deviate from the theme of sustainable development.

Piezoelectric materials are functional materials with a non-centrosymmetric structure, a class of intelligent functional materials that can spontaneously convert external mechanical energy into surface electrostatic charges [24,25]. Owing to the non-centrosymmetric crystal structure and internal atomic polarization displacement of piezoelectric materials, stable electrostatic charges can be continuously generated under dynamic pressure of airflow during the filtration process, without relying on external power supply or pre-charging treatment. This intrinsic piezoelectric effect effectively overcomes the problem of charge attenuation in conventional electret materials and establishes a synergistic filtration system combining physical interception and electrostatic adsorption, which significantly improves the capture efficiency of ultrafine particulate matters while maintaining low airflow resistance [26,27].

Poly(L-lactic acid) (PLLA), as a bio-based and biodegradable polymer, has demonstrated excellent application potential in multiple fields [28,29,30]. Especially in the field of air filtration, electrospun PLLA fiber membranes have received extensive research due to their piezoelectric properties, which can enhance the electrostatic adsorption capacity for ultrafine PMs [31,32,33,34]. For example, Nguyen et al. enhanced the piezoelectric performance of electrospun PLLA fibers by increasing their crystallinity and orientation, and thus successfully prepared membranes with excellent air filtration performance [34]. In our earlier work, a PLLA fiber membrane with multi-structured networks, which was welded from ribbon-like fibers and ultrafine nanofibers, was successfully prepared [31]. Thanks to this structure, the PLLA fiber membrane successfully balances the contradiction between filtration efficiency (>99.9% for PM_2.5_ and >99.5% for PM_0.3_) and pressure drop (~20 Pa). However, up to now, there have been few studies on the application of PLLA fiber membranes in air filtration under various harsh conditions, such as extreme temperatures and acidic and alkaline environments. The main reason for this is that the environmental stability of the PLLA material itself is not so great. Besides the complex process, enhancing its environmental stability through modification, doping and other methods may also affect its biodegradability and piezoelectric performance. It is well known that ultra-high molecular weight (UHMW) polymers usually have better environmental stability. UHMW polymers feature longer molecular chains, stronger interchain entanglement, lower end-group density, and more perfect crystalline regions [35,36,37], which can effectively block the erosion of chemical media and harsh environments and thus endow the materials with significantly improved chemical and environmental stability, such as the commonly used UHMW polyethylene at present [38,39]. However, due to excessive viscosity of their solutions, it usually increases the processing difficulty of fiber membranes.

In this work, firstly, UHMW PLLA fiber membranes were successfully prepared by using electro-blown spinning (EBS) technology. Under the combined action of the electric field force and the drawing force generated by high-pressure gas flow, a fluffy fiber membrane with a multi-scale structure was successfully prepared, and the spinning efficiency was greatly improved. Thanks to the piezoelectric properties of PLLA fibers and the fluffy structure of the film, the prepared UHMW PLLA fiber membrane exhibits excellent air filtration performance. The stability of the filtration performance of the fiber membrane under different environmental conditions was further evaluated, such as under high humidity, different temperatures, and acidic or alkaline environments.

## 2. Results and Discussion

**Figure 1:**
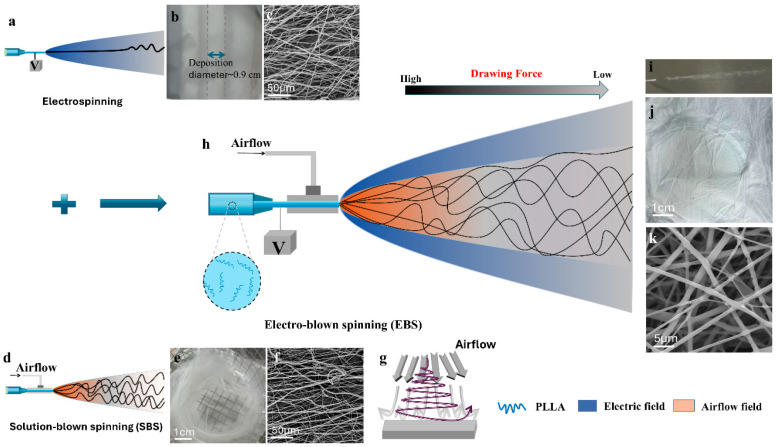
Schematic diagrams of the spinning process: (**a**) electrospinning, (**d**) solution-blown spinning (SBS), and (**h**) electro-blown spinning (EBS). (**b**) Optical and (**c**) scanning electron microscope (SEM) image of electrospun ultra-high molecular weight poly(L-lactic acid) (UHMW PLLA) fiber membrane. (**e**) Optical and (**f**) SEM image of UHMW PLLA three-dimensional fiber structure prepared through SBS technology. (**g**) Schematic diagram of the airflow colliding with the collector. (**i**) Optical image of the EBS process. (**j**) Optical and (**k**) SEM image of UHMW PLLA fiber membrane prepared through EBS technology.

Electrospinning and solution-blown spinning (SBS) are currently commonly used effective techniques for preparing micro-nano fibers [40,41,42]. The former mainly relies on the electric field force and electrostatic repulsion generated by the applied high voltage to prepare fibers, while the latter depends on the effect of pressurized gas. However, neither of these two techniques is suitable for preparing UHMW PLLA fiber membranes. When prepared through electrospinning technology, with a 1 wt% low-concentration solution, fibers with beads are prepared due to a lack of sufficient links in the molecular chains as shown in Figure S5a,d. Once the concentration is increased to 2 wt% or 3 wt%, uniform-structure bead-free fibers are obtained (Figure S5b,c and Figure 1c). However, due to the excessive viscosity of the solution at this time, as shown in Figure 1a, the jet is very stable and only slight whips occur when approaching the collector. The diameter of its deposition on the collector is only about ~1 cm (Figure 1b). This means that it is very difficult to prepare a relatively uniform fiber membrane. In addition, since the jet has not been sufficiently stretched, the diameter of the prepared fibers is generally at the micrometer level as shown in Figure S5e,f. There is another disadvantage, which is that due to the high viscosity of the solution, it is very easy to cause nozzle clogging, resulting in low spinning efficiency and material waste.

In the SBS process, a high pressurized gas stream is ejected and generates powerful shear force at the interface of the solution and gas. Although the viscosity of a 3 wt% solution is extremely high, the shear force can still overcome it to form stable jets. And under the action of the airflow, it is further stretched to form nanofibers as shown in Figure 1f. However, when the airflow encounters an obstacle, it is bound to reflect and rebound, thereby driving the fibers to rebound (Figure 1g). Eventually, it is easy to form a three-dimensional structure with obvious layering (Figure 1e) rather than a two-dimensional film that is convenient for application in the field of air filtration.

EBS is a hybrid technique that combines electrospinning and SBS [43,44]. As shown in Figure 1h, the shear force generated by high pressurized gas stream not only promotes the production of more jets and improves the spinning efficiency, but also solves the problem of insufficient stretching of the jet by the electric field force in the individual electrospinning process. Meanwhile, due to the existence of an electrostatic field between the nozzle and the collector, the effect of the airflow impact rebound is effectively suppressed, thereby solving the problem of easily forming a three-dimensional structure during the individual SBS process. As shown in Figure 1i and Video S1 in Supporting Information, smooth spinning can be carried out on a 3 wt% solution by using EBS technology. Two-dimensional UHMW PLLA fiber membranes composed of micro-nano fibers were successfully prepared as shown in Figure 1j,k. And the polymer feeding rate could reach 0.3 mL min^−1^, which is 20 times that of the electrospinning process.

**Figure 2:**
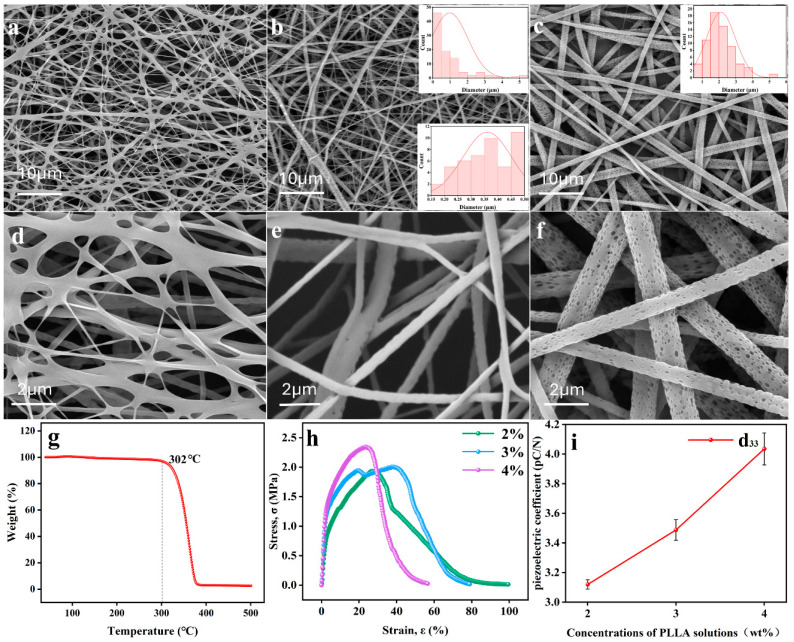
SEM images of UHMW PLLA fiber membranes prepared using solutions of different concentrations: (**a**) 2 wt%, (**b**) 3 wt%, and (**c**) 4 wt%. The insets of (**b**,**c**) are the corresponding distribution of fiber diameters in the figures. (**d**–**f**) Magnified partial images of Figure (**a**–**c**), respectively. (**g**) Thermal gravimetric analysis (TGA) curve of UHMW PLLA fiber membrane. (**h**) Tensile stress–strain curves of UHMW PLLA fiber membranes prepared using solutions of different concentrations. (**i**) Piezoelectric coefficients of UHMW PLLA fiber membranes prepared using solutions of different concentrations.

Then, the 2 wt% and 4 wt% solutions were spun respectively by using the EBS technology as shown in Figure 2a–f. For the 2 wt% solution, due to insufficient jet flight time, the solvent did not completely evaporate, and the incompletely solidified fibers collided with the collector, forming banded fibers (Figure 2a,d). For the 4 wt% solution, UHMW PLLA fiber membrane was successfully prepared, just like for the 3 wt% solution. Moreover, due to the extremely fast evaporation rate of the solvent dichloromethane (DCM), obvious holes appeared on the surface of fibers as shown in Figure 2b,c,e,f [45,46]. However, due to excessive viscosity of the 4 wt% solution, the diameters of fibers prepared using the current preparation parameters are mostly above 1 μm as shown in the inset of Figure 2c. Moreover, the needle is prone to clogging during the spinning process. By comparison, the fiber membrane prepared with the 3 wt% solution contains a large number of nanofibers and shows a distinct multiple structure composed of micrometer-level fibers and nanofibers (Figure 2b and insets).

It is well known that in the EBS process, the regulation of gas pressure, applied voltage, collection distance, feeding rate, and various environmental conditions, etc., can all affect the morphology of fibers [43,47]. However, this work aims to explore the feasibility of preparing UHMW PLLA nanofiber membranes and, on this basis, study the application of fiber membranes in the field of multiple environmental air filtration. And considering feasibility in actual production, relatively conventional and moderate spinning parameters were selected in this work, that is, not too high applied voltage (15 kV) and gas pressure (150 kPa). There was no attempt to explore conditions suitable for spinning solutions with higher concentrations (≥4 wt%) by regulating the spinning parameters.

All these fiber membranes have demonstrated excellent thermal stability and mechanical properties. As the thermal gravimetric analysis (TGA) curve has shown in Figure 2g, a fiber membrane begins to pyrolyze at around 300 °C and eventually completely generates small-molecule organic compounds, carbon dioxide, and water, etc. Fiber membranes prepared with 2 wt% and 3 wt% solutions exhibited greater tensile deformation, while those prepared with a 4 wt% solution had higher stress due to thicker and denser fibers (Figure 2h). Piezoelectric performance is an important factor in determining the electrostatic adsorption capacity of PLLA nanofiber membranes for ultrafine particles [34,48]. Although the d_33_ measured by the piezoelectric coefficient tester cannot truly reflect the piezoelectric properties of nanofibers, it can still prove that a fiber membrane has piezoelectricity. As shown in Figure 2i, due to reasons such as higher crystallinity and more uniform fiber morphology of fibers prepared from high-concentration solutions, fiber membranes prepared from 4 wt% solution exhibit a greater piezoelectric coefficient.

**Figure 3:**
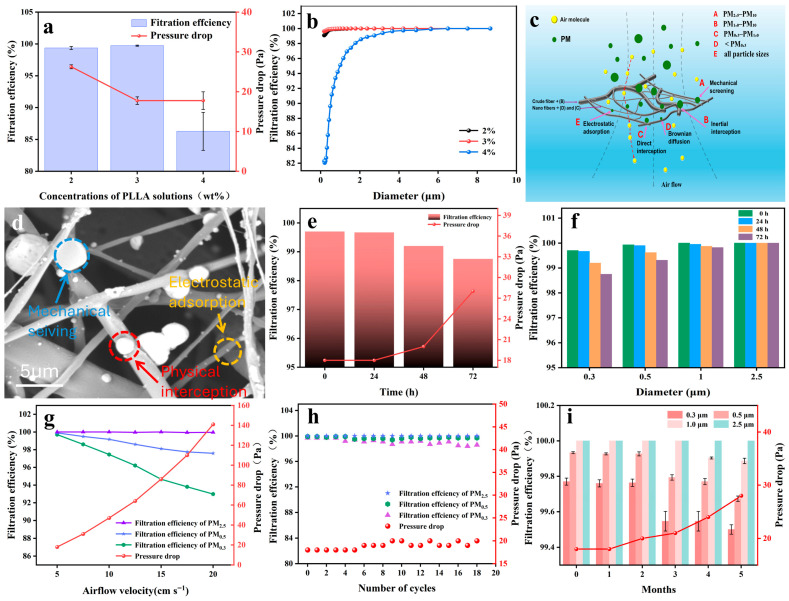
(**a**) Air filtration performance of UHMW PLLA fiber membranes prepared using solutions of different concentrations. (**b**) Filtration efficiency of UHMW PLLA fiber membranes prepared using solutions of different concentrations for different particle sizes of PMs. (**c**) Schematic diagram of the filtration mechanism of the UHMW PLLA fiber membrane. (**d**) A SEM image of the UHMW PLLA fiber membrane after filtration. (**e**) Changes in filtration performance and (**f**) changes in filtration efficiency for PMs of different particle sizes after a UHMW PLLA fiber membrane was placed in a saturated water vapor environment for different periods of time. (**g**) Filtration efficiency and pressure drop of the UHMW PLLA fiber membrane under various airflow velocities. (**h**) Cyclic stability of air filtration performance of the UHMW PLLA fiber membrane. (**i**) Time stability of air filtration performance of the UHMW PLLA fiber membrane.

The filtration performance of UHMW PLLA fiber membranes prepared from solutions of different concentrations is shown in Figure 3a. For the sake of comparison, the same amount of PLLA was maintained during the preparation of the three fiber membranes. As mentioned above, due to excessive viscosity of the 4 wt% solution, it is prone to clogging the needle tube under the current spinning parameters, resulting in solution waste. Therefore, filtration performance of the prepared fiber membrane is extremely unstable. Moreover, because some solution is wasted, the thickness of the fiber membrane is insufficient, thus filtration efficiency is relatively low.

As shown in Figure 2a,b, such nanofiber membranes with multiple structures prepared from 2 wt% and 3 wt% concentration solutions have been proven to typically possess excellent air filtration performance and to be able to effectively mediate the contradiction between filtration efficiency and pressure drop [11,31]. In this work, they also demonstrated excellent air filtration performance, especially UHMW PLLA fiber membranes prepared with a 3 wt% solution. They have demonstrated extremely high filtration efficiency for PM of different particle sizes, and even filtration efficiency for PM_0.3_ exceeds 99.8% as shown in Figure 3b and Figure S6. And at the same time, they have an ultra-low pressure drop of less than 20 Pa (Figure 3a). In addition, considering that the 3 wt% solution has higher efficiency in preparation of UHMW PLLA fiber membranes, all subsequent test analyses were conducted using samples prepared with this concentration of solution.

Generally speaking, the quality factor of air filtration membranes can be optimized by adjusting the base weight of the fiber membrane [49,50]. As shown in Figure S7a, with the reduction in the base weight of the fiber membrane, the pressure drop has been significantly decreased. Especially when the basis weight is reduced to 1.83 g m^−2^, the pressure drop is only about ~10 Pa, but at this time, its filtration efficiency still exceeds 99%, and even filtration efficiency for PM_0.3_ remains at around ~99% as shown in Figure S7b. Such excellent filtration performance has hardly been reported even in the currently widely studied fossil-derived polymer materials. Similarly, for PLLA-based air filtration membranes, the PLLA MSN membrane reported by Li achieved a maximum quality factor of 0.31 Pa^−1^ at a base weight of 2.8 g·m^−2^, whereas our membrane attained an excellent quality factor of 0.388 Pa^−1^ at a base weight of 2.45 g·m^−2^ [31]. Although air filtration performance of the UHMW PLLA fiber membrane is excellent, its thickness is relatively low, which increases the randomness in the preparation process. Therefore, in order to ensure the reliability of the subsequent test results, the fiber membrane with a base weight of 2.45 g m^−2^ is selected. This can ensure the uniformity of the fiber membrane and facilitate its stripping from the collection plate.

Extremely low pressure drop is mainly attributed to the existence of large pores, as shown in Figure 3c, through which air molecules can freely pass. The main reason for the formation of large pores lies in the reverse force (Figure 1g) formed by the impact of airflow on the collector during the EBS process. Although, as mentioned above, this force is suppressed by the electric field force and no longer forms a three-dimensional structure, and it becomes fluffier compared to the electrospun fiber membrane structure. In addition, coarser micron-sized fibers in the fiber membrane also form larger pores. Filtration mechanisms for PMs mainly include mechanical sieving, physical interception, and electrostatic adsorption (Figure 3c). Mechanical sieving relies on the geometric size effect of fiber membrane pores to directly intercept particles with diameters larger than the membrane pore size. Physical interception uses the effects of inertial collision, Brownian diffusion, and airflow bypass to cause submicron-sized ultrafine particles to deviate from the airflow streamline and adhere to the fiber surface. Electrostatic adsorption utilizes the Coulomb attraction generated by the electrostatic field on the fiber surface to actively capture tiny particles that are difficult to be intercepted by physical means, further enhancing the overall filtering effect [9,51,52]. As shown in Figure 3d, all of them can be found in the scanning electron microscope (SEM) images after the air filtration performance test. Electrostatic adsorption is the main mechanism for intercepting ultrafine PMs. The net charge excited by UHMW PLLA fiber membranes with piezoelectric properties during the air filtration process can effectively enhance the electrostatic adsorption effect. However, due to the inevitable generation of static electricity in the samples during the electrospinning process, in order to further determine the main source of the electrostatic adsorption effect during the filtration process, the UHMW PLLA fiber membrane was placed in a high-humidity environment for different periods of time to stand still. One of the purposes of this move was to conduct away the static electricity generated during the sample preparation process.

As shown in Figure 3e, after 72 h of placement, the filtration efficiency of the UHMW PLLA fiber membrane only slightly decreased and remained at around ~99%. Even the filtration efficiency for PM_0.3_ still exceeded 98.5% (Figure 3f). Pressure drop increased due to the condensation of water vapor on the surface of the fiber membrane. This indicates that the piezoelectric effect is the main factor for the excellent electrostatic adsorption of the fiber membranes. It also proves that the air filtration performance of UHMW PLLA fiber membranes has excellent moisture resistance and that it is suitable for applications in high-humidity environments. The aqueous solution was further replaced with a highly volatile concentrated hydrochloric acid (HCl, PH = 1) solution because an acidic environment has better electrical conductivity. As shown in Figure S8, similar results further demonstrate the reliability of the above conclusion.

To further evaluate the air filtration performance of the UHMW PLLA fiber membrane, it was tested at different airflow velocities. As shown in Figure 3g, filtration efficiency for PM_2.5_ has only decreased slightly when the airflow velocity increased from 5.33 cm s^−1^ to 20 cm s^−1^. But for ultrafine particles PM_0.3_, there was a relatively obvious decline, although it could still be maintained above ~92%. This is mainly because the UHMW PLLA fiber membrane is too fluffy, and when ultrafine particles pass through quickly, they are not fully captured. This problem should be solved by adjusting the thickness of the fiber membrane or regulating its fluffiness. In addition, the pressure drop increases linearly with the increase in airflow velocity. When the velocity reaches 20 cm s^−1^, it rises to ~140 Pa but still remains at a relatively low value. This trend is consistent with fluid dynamics principles, whereby higher gas flow velocities increase the resistance experienced by the gas phase passing through the membrane.

As shown in Figure 3h,i, the UHMW PLLA fiber membranes also demonstrated excellent cycling stability and time stability. Their interception capacity for particles of different sizes only showed a very slight decline after 18 cycles of filtration tests. The pressure drop also remained basically unchanged, staying below ~20 Pa (Figure 3h). After being placed under normal conditions for as long as five months, they can still maintain excellent filtration performance. The interception capacity for ultrafine PM_0.3_ remains above ~99%. Although the pressure drop has slightly increased, it still remains at an extremely low level below 30 Pa as shown in Figure 3i. This also indicates that the structure and performance of the UHMW PLLA fiber membranes is very stable under normal conditions.

To further evaluate the application potential of the UHMW PLLA fiber membrane, its dust-holding capacity was tested. As shown in Figure S9a,b, the surface of the UHMW PLLA fiber membrane was completely covered after continuous filtration for 13 h. During the continuous filtration process, its filtration efficiency remains basically unchanged (Figure S9c), while the pressure drop gradually increases in three stages (Figure S9d). Because fibers become coarser after adsorbing PMs, the pressure drop increases slowly within the first 3 h. Subsequently, as more and more PMs were adsorbed and intercepted through sieving, the holes became smaller and smaller, causing the pressure drop to increase at an accelerated rate. After continuous filtration for 10 h, the increase in pressure drop further accelerated. This is mainly because as the channels in the fiber membrane are blocked, more and more PMs deposit on the surface, and a dust cake layer begins to form. After continuous filtration for 13 h, the pressure drop rose to about ~50 Pa, which is still a very low value. At this point, the dust-holding capacity is as high as 19.04 g m^−2^, demonstrating the strong application potential of the UHMW PLLA fiber membrane.

The above results indicate that the prepared UHMW PLLA fiber membranes have excellent air filtration performance and demonstrate its application potential in this field. The following will further evaluate the stability of its air filtration performance under harsh conditions, such as acidity and alkalinity and high and low temperatures, and verify its potential for application in multiple environments.

To verify the improvement of the chemical resistance of UHMW PLLA fiber membranes, low-molecular-weight (LMW) PLLA fiber membranes were prepared using EBS technology with the same parameters for comparison. This molecular weight is currently the most commonly selected in research related to preparation of PLLA fiber membranes through electrospinning technology [31,34,53]. As shown in Figure S10a, due to low viscosity of the solution, a large number of spindle-shaped or coronal structures at the micrometer level appear in the LMW PLLA fiber membrane. Due to its unique structure, the LMW PLLA fiber membrane also demonstrates excellent air filtration performance, with filtration efficiency exceeding 99.5% and a pressure drop of only around ~20 Pa (Figure S11a,b). To verify their chemical resistance, first, spray bottles filled with HCl (PH = 1) or sodium hydroxide (NaOH, PH = 12) solutions of different PH values were evenly sprayed on their surfaces. After drying, air filtration performance tests were conducted. As shown in Figure S11a,b, air filtration performance of the LMW PLLA fiber membrane after treatment deteriorated significantly, filtration efficiency decreased markedly, and pressure drop rose sharply. This is mainly caused by the corrosion of the fiber membrane due to acidic and alkaline droplets as shown in Figure S10b,c.

**Figure 4:**
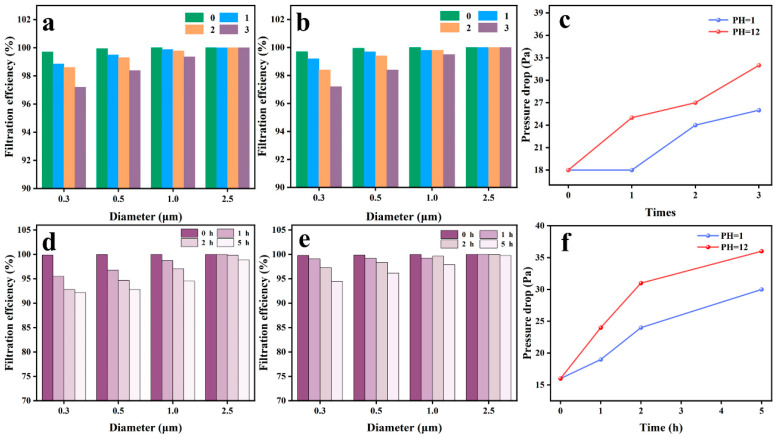
Chemical stability of the air filtration performance of UHMW PLLA fiber membranes. Changes in the filtration efficiency of UHMW PLLA fiber membranes for PMs of different particle sizes after being uniformly sprayed with (**a**) acidic or (**b**) alkaline solutions for different durations. (**c**) Changes in pressure drop of UHMW PLLA fiber membranes after being uniformly sprayed with acidic or alkaline solutions for different durations. Changes in filtration efficiency of UHMW PLLA fiber membranes for PMs of different particle sizes after soaking in (**d**) acidic or (**e**) alkaline solutions for different periods of time. (**f**) Changes in pressure drop of UHMW PLLA fiber membranes after soaking in acidic or alkaline solutions for different periods of time.

The UHMW PLLA fiber membranes that have undergone the same treatment look basically unchanged (Figure S10d–f), and their filtration performance has only declined (Figure S11a,b). They were further subjected to the same treatment twice, and air filtration performance tests were conducted after each treatment. As shown in Figure 4a,b, fiber membranes treated with acidic and alkaline solution jetting exhibited similar trends in filtration performance changes. The interception capacity for particles larger than PM_1.0_ remains basically unchanged, while it slightly decreases for smaller particle sizes of PMs. Despite this, filtration efficiency of the fiber membrane for PM_0.3_ can still be maintained at around ~97% after three treatments. The pressure drops all increased, among which the increase after alkaline solution treatment was even greater, reaching up to ~33 Pa (Figure 4c). This indicates that the air filtration performance of the prepared UHMW PLLA fiber membranes have relatively excellent chemical resistance.

To further verify chemical stability, the prepared UHMW PLLA fiber membranes were respectively immersed in the above-mentioned HCl and NaOH solutions. As shown in Figures S12 and S13, after being soaked for as long as 5 h, there was basically no obvious change in their appearance. Their filtration performance changes are basically the same as the trend after the above-mentioned solution spray treatment, but they are significantly more affected. Among them, the interception capacity for PM_2.5_ remained basically unchanged, but it had a significant impact on smaller particle sizes of PMs, especially PM_0.3_ (Figure 4d,e). However, their filtration efficiency is still above 90%. After soaking in HCl solution for 5 h, it drops to ~92%, while that of NaOH solution is ~94%. The pressure drop change is also similar to that after the spraying treatment and has not increased significantly due to long-term soaking as shown in Figure 4f. This further demonstrates excellent chemical stability of UHMW PLLA fiber membranes, laying a foundation for their practical application in complex and harsh environments.

**Figure 5:**
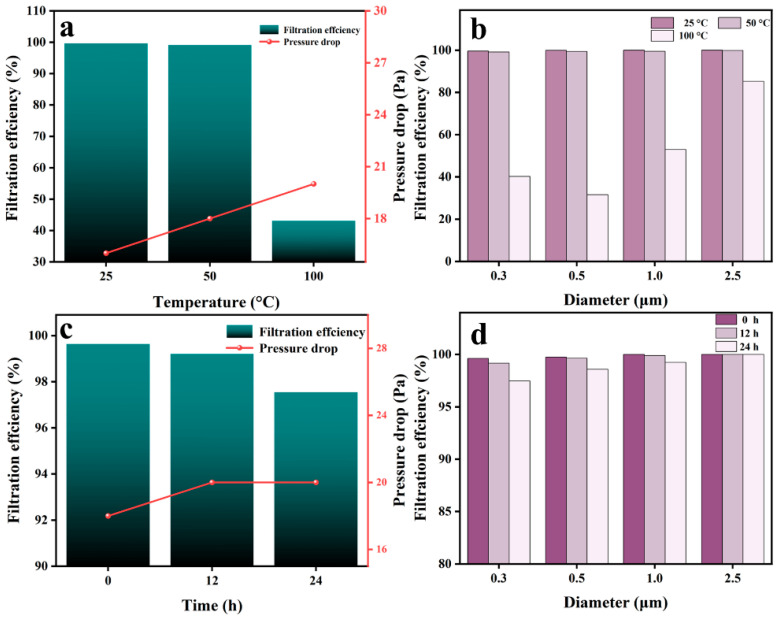
Temperature stability of air filtration performance of UHMW PLLA fiber membranes. (**a**) Changes in air filtration performance of the UHMW PLLA fiber membrane after high-temperature treatment. (**b**) Changes in filtration efficiency of the UHMW PLLA fiber membrane for PMs of different particle sizes after high-temperature treatment. (**c**) Changes in air filtration performance of the UHMW PLLA fiber membrane after being frozen at low temperatures for different periods of time. (**d**) Changes in filtration efficiency of the UHMW PLLA fiber membrane for PMs of different particle sizes after being frozen at low temperatures for different periods of time.

To evaluate the stability of UHMW PLLA fiber membrane air filtration performance at high temperatures, it was first heated to 50 °C in a muffle furnace and maintained at this temperature for 2 h. As shown in Figure 5a,b, its air filtration performance is almost the same as that at ambient temperature, with only a slight increase in pressure drop. This indicates that it is extremely stable within 50 °C. The temperature was further raised to 100 °C and maintained for 2 h. Filtration efficiency has decreased significantly as shown in Figure 5a. Although filtration efficiency for PM_2.5_ can still be maintained at around ~80%, filtration efficiency for ultrafine PMs with particle size below PM_0.5_ has dropped below ~40% (Figure 5b). At this point, by observing the SEM image as shown in Figure S14, almost no obvious changes in structure and fiber morphology can be seen. The only slight change in pressure drop can also confirm this (Figure 5a). Meanwhile, the piezoelectric coefficient of the fiber membrane is almost undetectable when measured using a piezoelectric coefficient meter, which indicates that the sharp decline in filtration efficiency is caused by the deterioration of piezoelectric performance. This once again confirms that the superior interception ability of UHMW PLLA fiber membranes for ultrafine PMs mainly stems from its excellent piezoelectric performance.

Temperature stability is also an important guarantee for the practical application of air filtration fiber membranes. As mentioned above, excellent electrostatic adsorption capacity derived from the piezoelectric properties of the material itself is an important reason for the outstanding filtration performance of UHMW PLLA fiber membranes. The piezoelectricity of PLLA originates from the rotation of carbonyl groups caused by shear stress applied to the molecular chain. Therefore, its piezoelectric performance is closely related to the order of molecular chain arrangement and the crystal phase structure [34,54]. The glass transition temperature of PLLA is approximately 60 °C. Beyond this temperature, the movement ability of the molecular chains significantly increases, which may break its ordered structure or lead to a transformation in the crystal phase structure. This leads to a reduction or disappearance of piezoelectric performance. In addition, heating in the air may also intensify oxidation reactions, leading to the breakage or decomposition of molecular chains, thereby affecting piezoelectric performance.

The stability of the UHMW PLLA fiber membrane air filtration performance at low temperatures was also evaluated. The prepared membrane was placed in a freezer at −19 °C for freezing. After 12 h and 24 h, it was taken out and tested for filtration performance. Just as after high-temperature treatment, freezing did not cause obvious changes in the structure and morphology of the fiber membrane (Figure S15). And as shown in Figure 5c,d, after 24 h of freezing, the filtration performance of the fiber membrane remained at around ~98%, and this slight decline was mainly caused by ultrafine PM_0.3_ (Figure 5d). The above results indicate that the air filtration performance of UHMW PLLA fiber membranes can maintain long-term stability within the range of −19 to 50 °C, enhancing their application potential in real life.

Finally, the biodegradability of the UHMW PLLA fiber membrane was verified. At the end of October, the fiber membrane was buried in the soil beside a landscape pool on campus without any other treatment. After about two months, right after a recent rain, when the soil was dug open, the fiber membrane was almost invisible, and only slightly whitened soil as shown in Figure S16 could be seen. This indicates that PLLA can naturally decompose in an environment where only moisture, microorganisms, etc., are present.

## 3. Conclusions

In summary, the drawing force generated by the high-pressure gas flow not only helps solve the problem of insufficient jet stretching in the individual electrospinning process, but also effectively improves spinning efficiency. At the same time, the existence of the electric field force effectively suppresses the rebound effect caused by the airflow impact on the collector. Based on this, UHMW PLLA fiber membranes were successfully and efficiently prepared by using hybrid EBS technology. This also provides a new approach to preparation of other UHMW polymer nanofibers.

Thanks to the excellent electrostatic adsorption capacity brought by the piezoelectric properties of the UHMW PLLA prepared fiber membrane and its fluffy multi-scale structure, it demonstrates outstanding air filtration performance. The contradiction between filtration efficiency (>99.8% for PM_0.3_) and pressure drop (~16 Pa) has been successfully balanced. After further optimization, while maintaining a filtration efficiency of over 99%, its pressure drop can be as low as 10 Pa. Its air filtration performance even surpasses that of fossil-derived polymer materials that have been widely studied at present. It also demonstrated excellent moisture resistance, long-term stability, and dust-holding capacity. Especially compared with low molecular weight PLLA fiber membranes, its air filtration performance demonstrates excellent chemical stability. Meanwhile, its temperature stability can also meet the needs of most scenarios in life. This ensures the feasibility of the practical application of sustainable material PLLA in the field of air filtration.

Due to it being a bio-based material with biodegradability, the excellent air filtration performance of UHMW PLLA fiber membranes can effectively reduce energy consumption during use, thus achieving sustainable development throughout the material’s entire life cycle. This might also provide some help and reference for solving global problems.

## Data Availability

The original contributions presented in this study are included in the article/Supplementary Materials. Further inquiries can be directed to the corresponding authors.

## Supplementary Materials

The following supporting information can be downloaded at: https://www.mdpi.com/article/10.3390/nano16100604/s1. Reference [55] is cited in the Supplementary Materials.
