# Supplementary material for: Electro-Blown Spun Ultra-High Molecular Weight Poly(L-Lactic Acid) Fibrous Membranes for High-Efficiency Air Filtration in Multiple Environments
Source: Nanomaterials (Basel). 2026 May 14;16(10):604. doi: 10.3390/nano16100604 (PMC13209628; doi:10.3390/nano16100604)
Supplement: Supplementary file 1 [file nanomaterials-16-00604-s001.zip › nanomaterials-4300021-supplementary.pdf]

Supporting Information

Experimental Section

1. Materials

Two types of Poly(L-lactic acid) (PLLA) with different molecular weights were purchased from Daigang Biodegradable Materials Co., Ltd. (Jinan, China). The one with a molecular weight of 1534000 was defined as ultra-high molecular weight PLLA (UHMW PLLA), and the molecular weight of 674000 was designated as low molecular weight (LMW) PLLA. Dichloromethane (DCM, ≥ 99.5%), sodium hydroxide (NaOH, 99%), and hydrochloric acid (HCl, 38%) were purchased from SinopHarm Chemical Reagent Co., Ltd. (Beijing, China).

2. Solution preparation

UHMW PLLA with different masses (0.2 g, 0.3 g, and 0.4 g) was added to 10 mL of dichloromethane (DCM), respectively. Subsequently, the mixtures were subjected to water bath heating at 40 °C with magnetic stirring until complete dissolution, yielding UHMW PLLA solutions with mass fractions of 2 wt%, 3 wt%, and 4 wt%, respectively. The 3 wt % LMW PLLA solution was also prepared in the same way.

A 150 mL hydrochloric acid (HCl) solution with pH = 1 was prepared by mixing 1.2 mL of concentrated HCl (mass fraction: 38%) with 148 mL of deionized water.

Similarly, a 150 mL sodium hydroxide (NaOH) solution with pH = 12 was obtained by dissolving 0.6 g of NaOH (purity: 99%) in 150 mL of deionized water.

3. Preparation of PLLA fiber membranes

Preparation of UHMW PLLA fiber membranes by Electrospinning

The UHMW PLLA fiber membrane was prepared by using the self-made electrospinning device. The solution feeding rate was 15 μL min^{-1} and the collection distance is kept at 15 cm. The applied voltage is kept at 15 kV. A rotating drum wrapped in aluminum foil was employed as the collector, with its rotational speed set at 300 r min^{-1}. The preparation process was conducted in a 20 ± 5% relative humidity atmosphere at ambient temperature.

Preparation of UHMW PLLA fiber membranes by Solution-blown spinning (SBS)

The prepared solution was loaded into a 10 mL plastic syringe fitted to a SBS apparatus (Model KJ-Z1, Qingdao Jinuo Robot Co., Ltd., China). A wire-framed collection cage was employed as the fiber collection device. The solution was extruded through the syringe needle tip at a feeding rate of 0.3 mL min^{-1} using a syringe pump. The compressed air pressure was set at 200 kPa, and the distance between the needle tip and the collection cage was fixed at 30 cm. The entire spinning process was conducted under ambient temperature conditions with a relative humidity of 20 ± 5%.

Preparation of UHMW and LMW PLLA fiber membranes by Electro-blown spinning (EBS)

The home-made EBS device was used to prepare ultra-high molecular weight fiber membranes. The voltage supply device was connected to the front end of the spinneret, while a sleeve for high-speed airflow delivery was fitted over the rear end of the spinneret, subsequently, the spinning syringe was mounted on the syringe pump.

The solution feeding rate was 0.3 mL min^{-1}. The collection device was made by closely bonding silicone oil paper and tin foil paper together, with a grounding device in the middle. The distance between the needle tip and the collection distance was 15 cm. The applied voltage is maintained at 15 kV, while the compressed air pressure is set to 150 kPa.

4. Characterization

The microscopic morphologies of the prepared fibrous membranes were characterized using a field-emission scanning electron microscope (FE-SEM, Model: PhenomPro, Phenom-World, The Netherlands). The piezoelectric coefficient of the PLLA membranes was determined with the piezoelectric coefficient tester (Model: ZJ-3A, Beijing Jingkezhi-chuang Technology Development Co., Ltd., China). The fiber diameters were measured via image analysis software (ImageJ, Version 1.54f, National Institutes of Health, USA). Tensile tests were carried out on an electronic universal testing machine (Shimadzu-AGS-X50) equipped with a 5000 N load cell. Thermogravimetric (TG) analysis was performed using a thermogravimetric-differential scanning calorimetry (TG-DSC) instrument (Model: TGA5500, USA).

Mechanical property characterization

Tensile tests were performed using an electronic universal testing machine equipped with a 5000 N force sensor; prior to testing, UHMW PLLA fibrous membrane specimens were cut into square shapes with a dimension of 20 mm × 20 mm and a thickness of 30 μm, and the testing parameters were set as follows: the crosshead speed was fixed at 15 mm min^{-1} and the gauge length was adjusted to 50 mm. For specimen mounting, one end of the UHMW PLLA fibrous membrane was clamped in the upper fixture of the testing machine while the other end was secured in the lower fixture, with clamping adjusted to ensure stability and alignment without slippage during the test. The tensile test was then initiated, and the measurement automatically ceased when the force sensor detected specimen fracture. Each test was repeated three times with parallel specimens to ensure data reliability, and the final results were reported as the average value of the three replicate measurements.

Thermogravimetric analysis test

Thermogravimetric (TG) analysis was performed using a thermogravimetric-differential scanning calorimetry (TG-DSC) instrument. The 3 wt% UHMW PLLA fiber membrane was cut into small square specimens with dimensions of 3 mm × 3 mm and a thickness of 30 μm. Each specimen was placed in an aluminum crucible, and the test was conducted under a nitrogen atmosphere to eliminate oxidative interference. The temperature program was set to ramp from room temperature to 800 °C at a constant heating rate of 10 °C min^{-1}. After the test, the mass of the crucible containing the residual char was compared with that of the empty aluminum crucible. No

measurable mass difference was detected, confirming that the UHMW PLLA fiber membrane specimens had undergone complete combustion.

5. Filtration performance test

The filtration efficiency and pressure drop of the UHMW PLLA fiber membranes were measured using the Palas MFP 3000 filter test bench (Fig. S1). The effective filtration area was 100 cm^2, and the solid potassium chloride particles produced by the atomizer were pumped by the air pump at a controlled flow rate of 5.33 to 20 cm s^{-1}. Conveyed through the test filter. These particulate matters (PMs) exhibited a mean diameter ranging from 0.225 to 10 μm. The size distribution of aerosol particles within the instrument during the filtration test is presented in Fig. S2. The filtration test apparatus was equipped with an electrostatic aerosol neutralizer to eliminate the surface charges of the particulate matters. Two laser particle counters were employed to quantify the particle concentrations upstream and downstream of the filter membrane, so as to evaluate the filtration efficiency of the sample; meanwhile, a differential pressure sensor was utilized to measure the pressure difference across the filter membrane for the assessment of pressure drop.

During the test, for each prepared fiber membrane (with dimensions of 35 cm × 20 cm), at least three distinct regions with an area of 100 cm^2 were selected for measurement, and the results were averaged to ensure data reliability. Unless otherwise specified, the test airflow velocity was set at 5.33 $cm\cdot s^{-1}$. This parameter was selected in accordance with the European standard (EN779: 2012) and the American standard

(IEST-RP-CC52.2-2007), which are widely recognized as the industrial testing standards for air filters. Furthermore, this airflow velocity of 5.33 $cm \cdot s^{-1}$ has been widely adopted by most global air filter manufacturers and in relevant published literature for evaluating filter performance. The relative humidity was maintained at 30% throughout the test.

In addition, the quality factor (QF), as an indicator to evaluate the filtration capacity of air filters based on their removal efficiency and air resistance, can be defined by the formula: $QF = -\ln(1-\eta)/\Delta p$ in which the η is the removal efficiency and the Δp is the pressure drop.[1]

Moisture resistance test

As shown in Fig. S3a,150 mL of distilled water was added to the bottom of a clean beaker. Subsequently, a piece of UHMW PLLA fibrous membrane was secured at the central position inside the beaker by using adhesive tape. The opening of the beaker was tightly sealed with parafilm to establish a sealed environment. Every 24 h, the fiber membrane is taken out for air filtration performance testing.

Air filtration performance test under acidic atmosphere

As shown in Fig. S3b, 150 mL of HCl solution was added to the bottom of a clean beaker. Subsequently, a piece of UHMW PLLA fibrous membrane was secured at the central position inside the beaker by means of adhesive tape. The opening of the beaker

was tightly sealed with parafilm to form a sealed environment. Every 24 h, the fiber membrane is taken out for air filtration performance testing.

Chemical stability test (Spray method)

Two LMW PLLA fibrous membranes were prepared, one was uniformly sprayed with HCl solution and the other with NaOH solution via a spray bottle. Throughout the entire spraying process, ensure that the film surface is completely and evenly covered. The two samples were then placed under identical environmental conditions and air-dried for 6 h, followed by the measurement of air filtration performance.

The two UHMW PLLA fiber membranes were treated in the same way. To further verify the stability of its air filtration performance, the same process was carried out three times.

Chemical stability test (Immersion method)

As shown in Fig. S4, two UHMW PLLA fibrous membranes were separately immersed in HCl and NaOH solutions, with full and uniform submersion ensured. After soaking for 1 h, they were taken out separately, air-dried and subjected to air filtration performance tests. Then, they were once again immersed in the solution for 1 h. After that, they were taken out respectively, air-dried and tested. Ultimately, the UHMW PLLA fiber membranes were soaked again for up to 3 h and then air-dried and subjected to performance tests. During the soaking period, to ensure the relatively stable concentration of the solutions, the Petri dish should always be covered with a lid.

Temperature stability test（High-temperature）

Lay the UHMW PLLA fiber membrane flat in the muffle furnace, set the temperature to 50 °C, with a heating time of 1 h and a holding time of 2 h. After the temperature drops to room temperature, take out the sample and conduct an air filtration performance test. Then put the sample back into the muffle furnace again, set the temperature to 100 °C, with a heating time of 1 h and a holding time of 2 h. After the holding time is over, cool it to room temperature, take out the sample and test the air filtration performance again.

Temperature stability test（Low-temperature）

Freeze the UHMW PLLA fiber membrane in a refrigerator at a temperature of -19 °C. Every 12 h, take out the sample and immediately conduct air filtration performance tests.

Porosity testing and calculation

The measurement using a precision balance yields W = 2.45 g·m^{-2}, The thickness was measured to be t = 30.2 μm using a thickness gauge. Its calculation formula is[2]

$$Apparent\ density(\partial_n) = \frac{w}{\pi \cdot \frac{\varphi^2}{4} \cdot t} \tag{S1}$$

$$Apparent\ porosity(\alpha) = \left(1 - \frac{\partial n}{Bulk\ density\ of\ PLLA}\right) \times 100\% \tag{S2}$$

$$u(p) = \sqrt{[(\partial P/\partial t)^2 \cdot u(t)^2 + (\partial P/\partial w)^2 \cdot u(w)^2]} \tag{S3}$$

The calculated porosity is P ≈ 93.5%. The corresponding measurement uncertainties are u(t) = 1.5 μm, u(W) = 0.06 g·m⁻², and u(P) ≈ 0.6%, so the porosity is determined as P = 93.5% ± 0.6%.

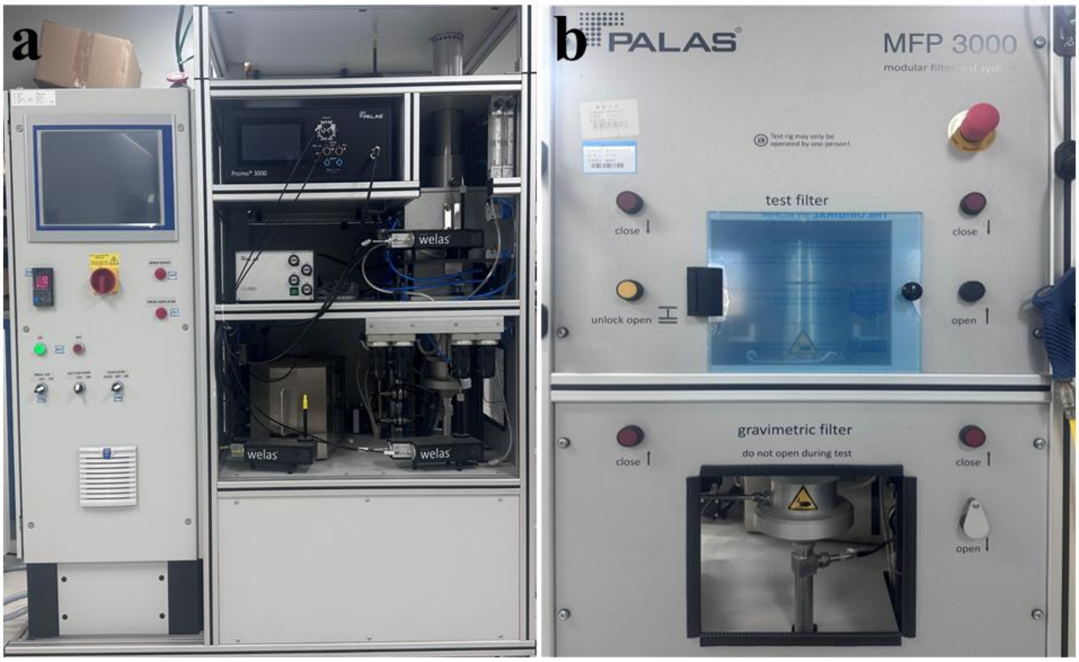

Figure S1. (a) The front optical image of PALAS MFP3000. (b) The side optical image of PALAS MFP3000.

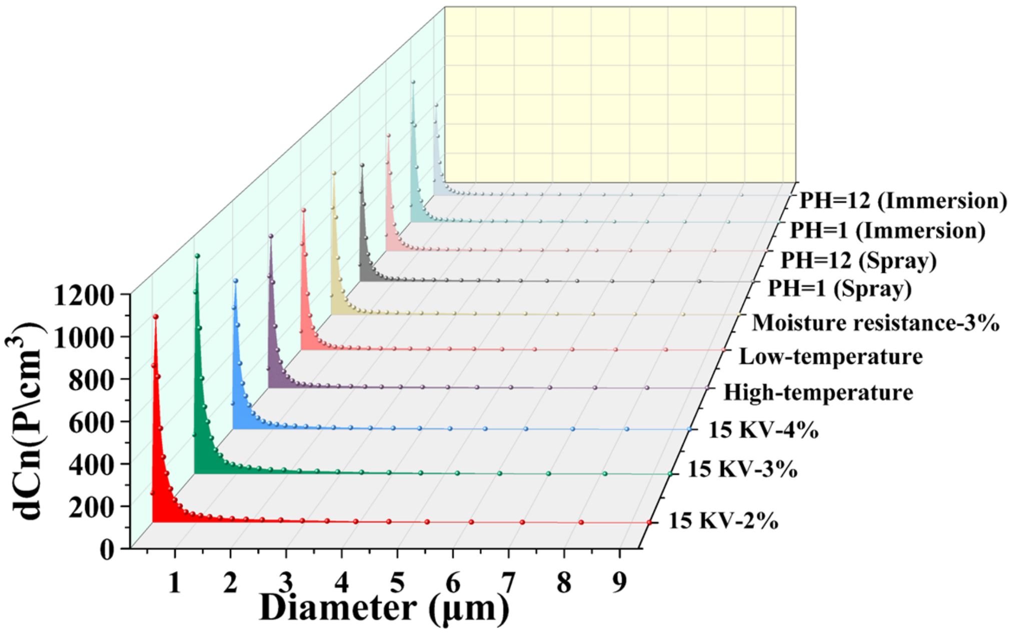

Figure S2. Particle size distribution during filtration efficiency testing of various UHMW PLLA fiber membranes using the PALAS MFP 3000 filter material test bench.

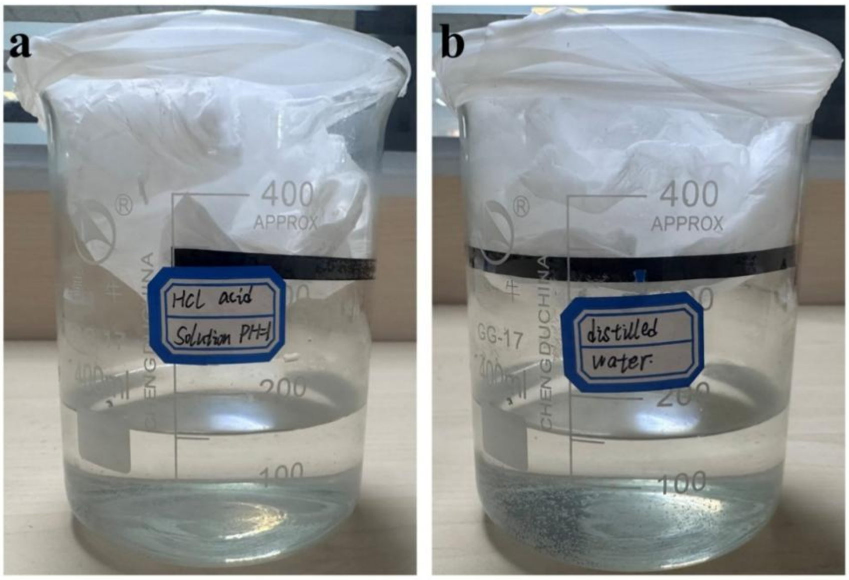

Figure S3. UHMW PLLA fiber membranes were respectively placed in (a) a saturated humidity environment and (b) an acidic environment.

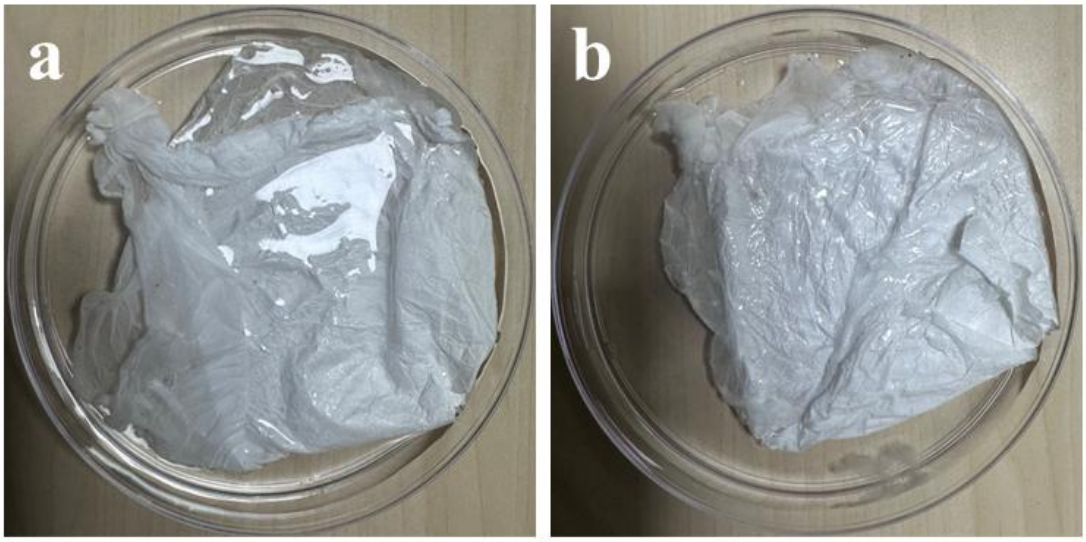

Figure S4. UHMW PLLA fiber membranes were respectively immersed in (a) acidic and (b) alkaline solutions.

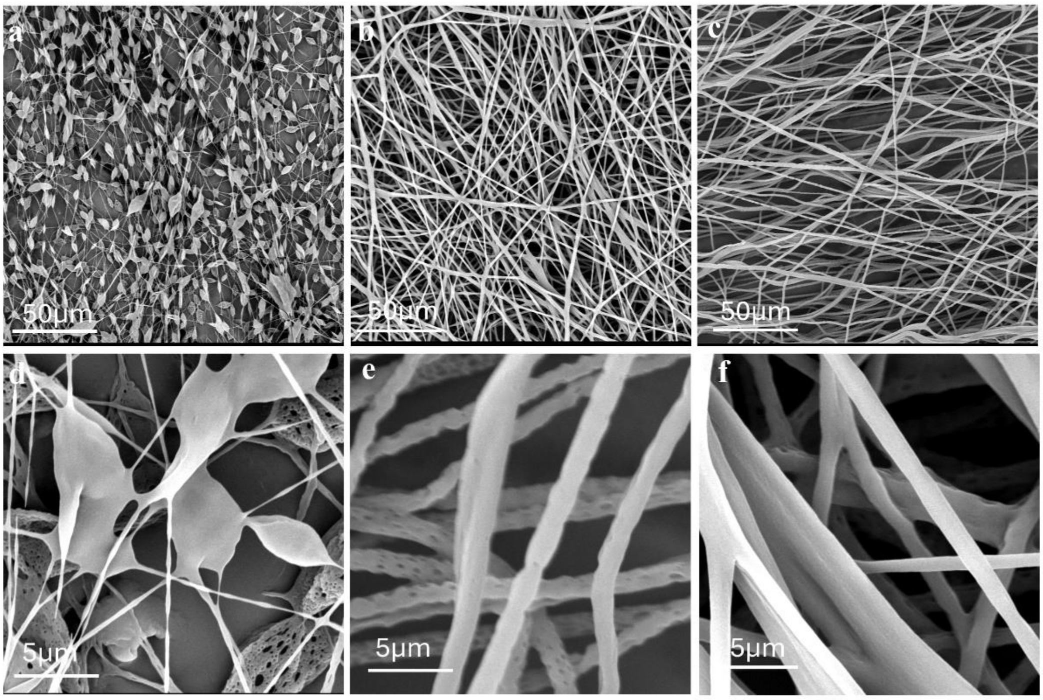

Figure S5. SEM images of UHMW PLLA electrospun fiber membranes prepared using solutions of different concentrations (a) 1 wt%, (b) 2 wt%, and (c) 3 wt%. (d)-(f) The magnified partial images of Fig.(a)-(c), respectively.

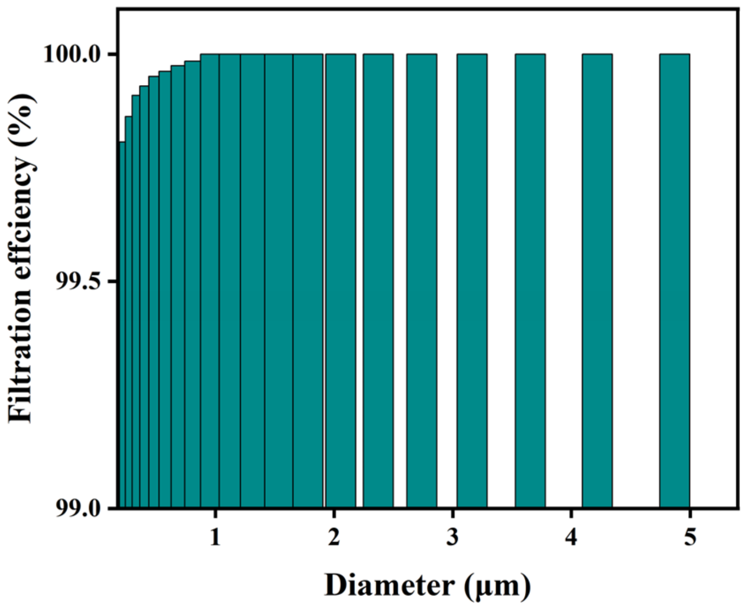

Figure S6. The filtration efficiency of UHMW PLLA fiber membranes for PMs of different particle sizes.

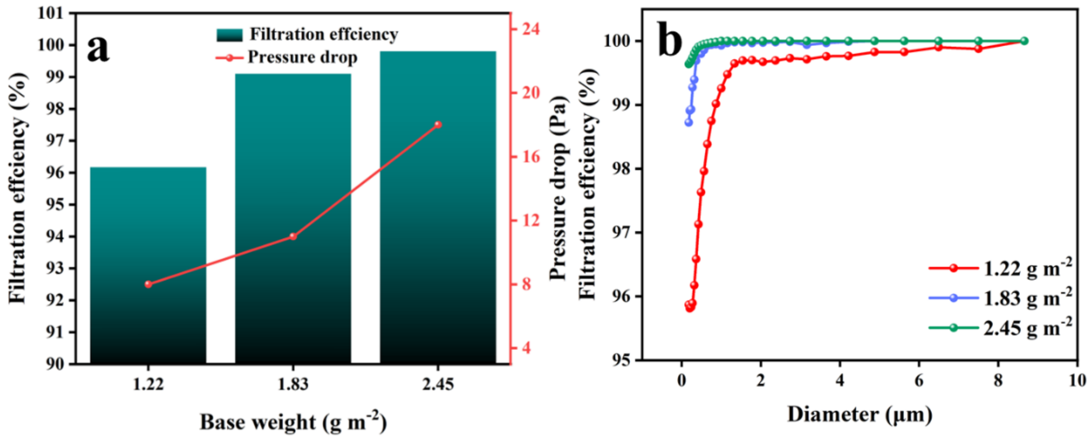

Figure S7. (a) The air filtration performance of UHMW PLLA fiber membranes prepared using solutions with different basis weights. (b) Filtration efficiency of UHMW PLLA fiber membranes with different basis weights for different particle sizes of PMs.

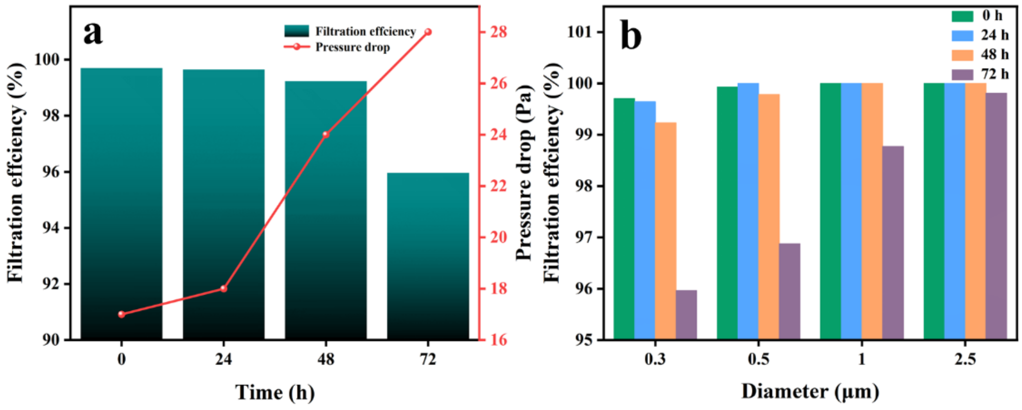

Figure S8. (a) The changes in filtration performance and (b) the changes in filtration efficiency for PMs of different particle sizes after UHMW PLLA fiber membrane was placed in an acidic environment for different periods of time.

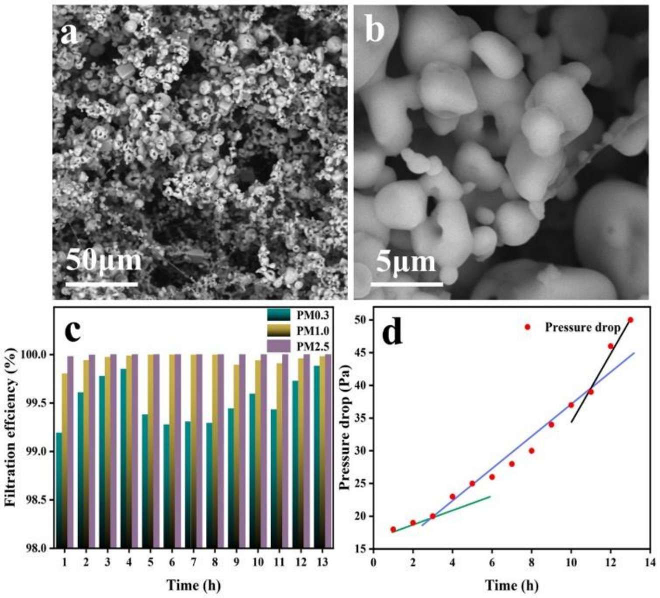

Figure S9. (a) SEM image of the UHMW MSN membrane after continuous filtration for 13 h. (b) The magnified partial images of Fig. (a). The variation of (c) filtration efficiency and (d) pressure drop of the UHMW MSN membrane with filtration time.

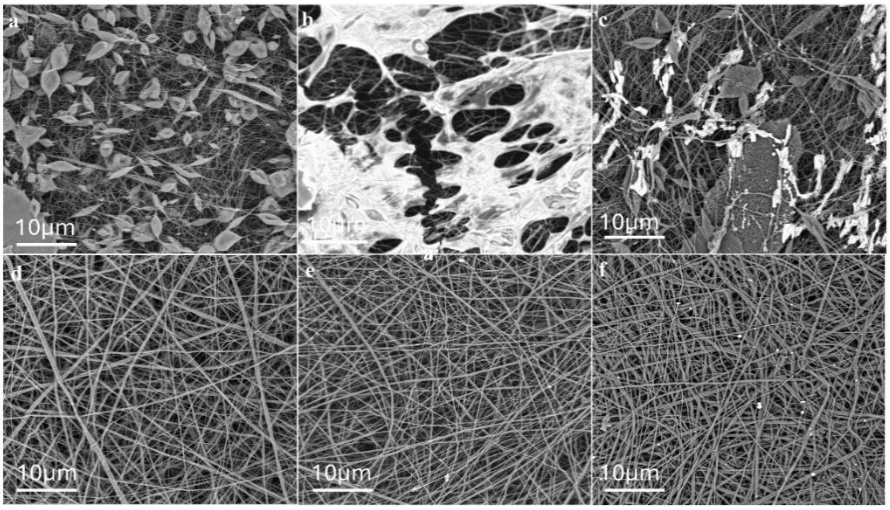

Figure S10. SEM images of the prepared LMW PLLA fiber membranes (a) without treatment, (b) after spraying with HCl solution, and (c) after spraying with NaOH solution. SEM images of the prepared UHMW PLLA fiber membranes (d) without treatment, (e) after spraying with HCl solution, and (f) after spraying with NaOH solution.

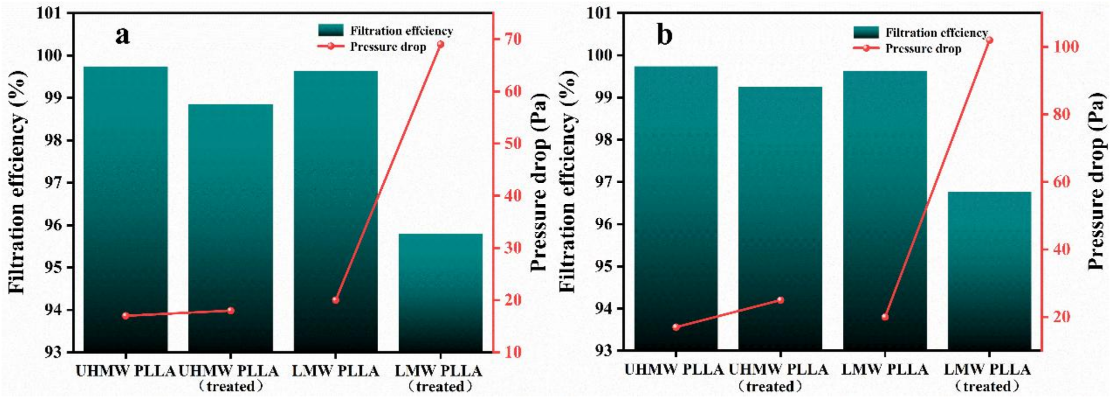

Figure S11. Filtration performance changes of UHMW PLLA and LMW PLLA fiber membranes after spraying with (a) HCl solution and (b) NaOH solution.

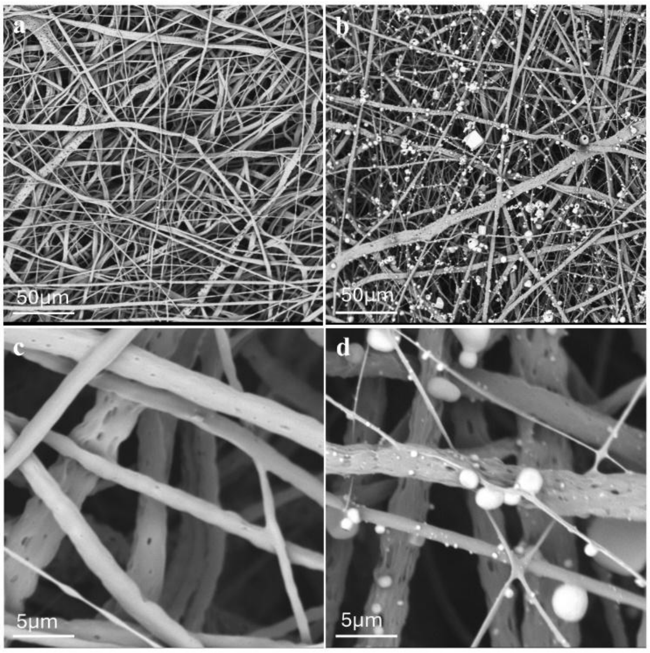

Figure S12. SEM images of the prepared UHMW PLLA fiber membranes (a) without treatment, (b) after immersion in HCl solution for 5 h. (c) - (d) The magnified partial images of Fig.(a) - (b), respectively.

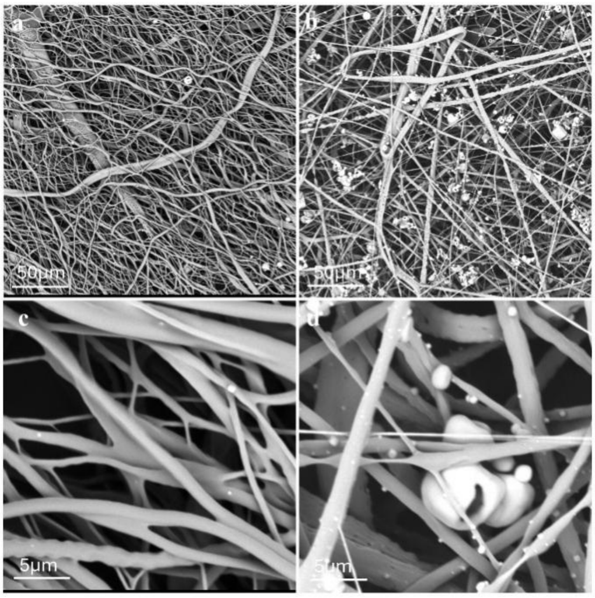

Figure S13. SEM images of the prepared UHMW PLLA fiber membranes (a) without treatment, (b) after immersion in NaOH solution for 5 h. (c) - (d) The magnified partial images of Fig.(a) - (b), respectively.

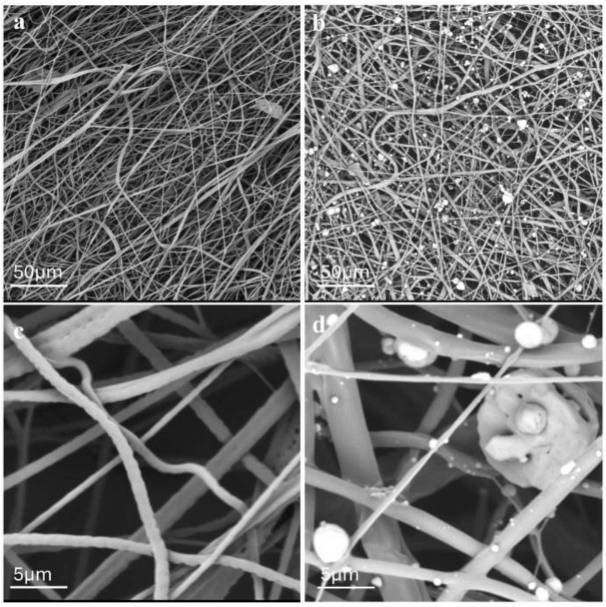

Figure S14. SEM images of the prepared UHMW PLLA fiber membranes (a) without treatment, (b) after being placed in a high-temperature environment of 100 °C for 2 h. (c) - (d) The magnified partial images of Fig.(a) - (b), respectively.

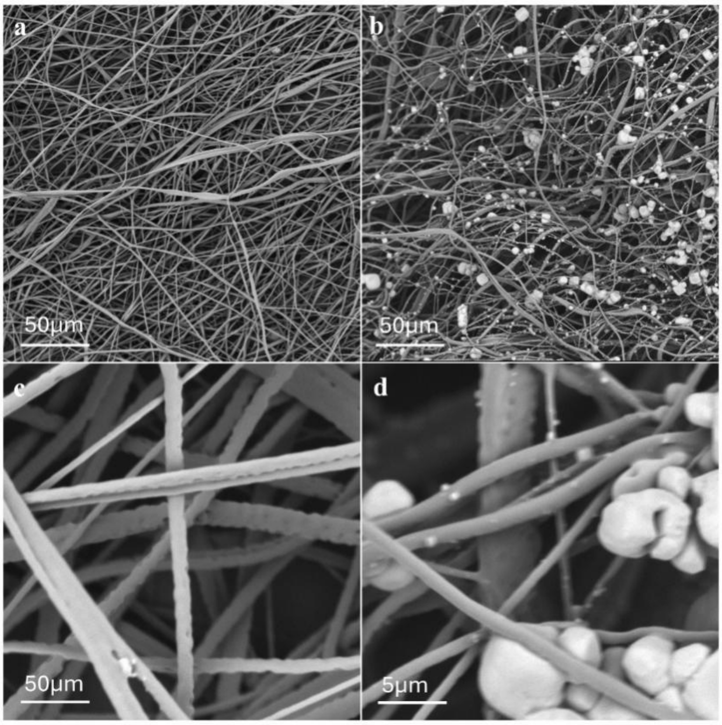

Figure S15. SEM images of the prepared UHMW PLLA fiber membranes (a) without treatment, (b) after being frozen at -19 °C for 24 h. (c) - (d) The magnified partial images of Fig. (a) - (b), respectively.

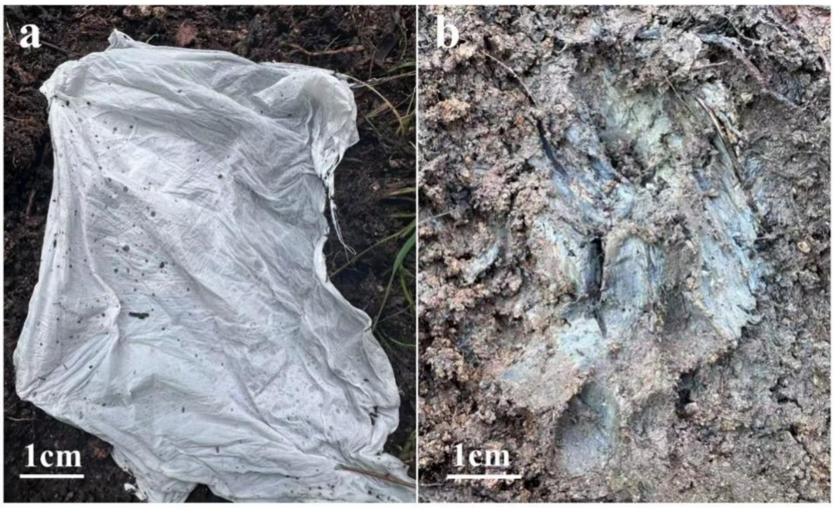

Figure S16. Optical image of (a) the prepared UHMW PLLA fiber membrane and (b) two months after it was buried in the soil.

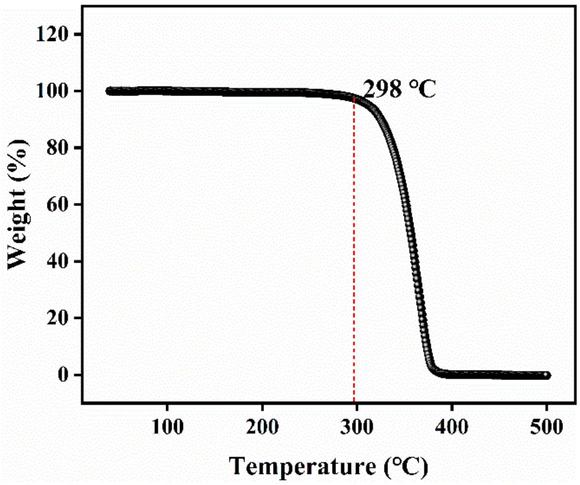

Figure S17. The thermal gravimetric analysis (TGA) curve of UHMW PLLA fiber membrane.

1. Li L, Gao Y, Nie G, et al. Biodegradable Poly (L-Lactic acid) Fibrous Membrane with Ribbon-Structured Fibers and Ultrafine Nanofibers Enhances Air Filtration Performance. *Small.* Nov 2024;20(44).
2. Naragund VS, Panda PK. Electrospun polyacrylonitrile nanofiber membranes for air filtration application. *International Journal of Environmental Science and Technology.* Oct 2022;19(10):10233-10244.
